# Established renal markers for diagnosis and prognosis in acutely decompensated heart failure

**DOI:** 10.1186/2197-425X-3-S1-A259

**Published:** 2015-10-01

**Authors:** M Brand, M Schwiede, H-J Trappe, M Christ, L Maier, A Luchner, CG Jungbauer

**Affiliations:** Marienhospital Herne - Ruhr University Bochum, Herne, Germany; University Medical Center Regensburg, Regensburg, Germany

## Objectives

Patients with acutely decompensated heart failure suffer often from deterioration of renal function, the so-called cardiorenal syndrome (CRS). This prospective study assessed whether established renal markers are relevant for diagnosis and establishment of prognosis in acute heart failure.

## Methods

The classic proteinuria markers - protein, albumin, IgG and alpha-1-microglobulin - were assessed from urine samples of 58 patients with acutely decompensated heart failure at admission, at day 2, at middle of therapy and at discharge. The serum levels of NT-proBNP, creatinine and cystatin C were acquired in the same way. The development of acute kidney injury (AKI) was analysed. Outcome parameters were all-cause mortality and a combined endpoint of death and rehospitalisation for congestive heart failure. Patients were followed for a median duration of 644 days (IQR 316, 837 days).

A Cox regression analysis including EF, occurrence of acute kidney injury, NYHA stage >2, BMI, age, serum creatinine and respectively one of the markers was performed to identify independent predictors. Independent predictors were further evaluated with ROC-curve analysis.

## Results

23 patients (40%) suffered from AKI. In patients without AKI a decrease in protein, albumin and IgG concentrations was observed during recompensation compared between the different sampling time points and day 1 (each p < 0.05). This decrease was most pronounced for albumin with a stepwise decrease over time. In contrast, there was no significant decrease in proteinuria in patients with AKI (each p= n.s.).

A total of 22 deaths were observed and 34 patients reached the combined endpoint.

At admission NT-proBNP and serum creatinine were significant predictors for mortality. Discharge levels of urinary protein and a1-microglobulin as well as NT-proBNP were significant predictors for all-cause mortality (each p < 0.05). Admission cystatin C performed as significant predictor for mortality as well as the combined endpoint (p < 0.05). In the Cox regression analysis admission cystatin C and a1-microglobulin at discharge performed as independent predictors for all-cause mortality, admission cystatin C also for the combined endpoint (each p < 0.05). Admission cystatin C (AUC of 0.68 for both endpoints) and discharge a1-microglobulin (AUC 0.69 for all-cause mortality) showed satisfying predictive values in the ROC curve analysis.

## Conclusions

Patients with AKI don´t show a significant decrease of urinary protein concentrations over time. Cystatin C at admission and a1-microglobulin at discharge offer important prognostic information in patients with acutely decompensated heart failure, superior to the established renal function parameters creatinine and eGFR. Therefore, renal function plays an important role for risk stratification and establishment of prognosis in cardiorenal syndrome.

